# Transdermal iontophoresis versus high power pain threshold ultrasound in Mechanical Neck Pain: a randomized controlled trial

**DOI:** 10.1186/s13018-024-05078-z

**Published:** 2024-10-15

**Authors:** Nouran A. Ibrahim, Hend A. Hamdy, Rana Hesham Mohamed Elbanna, Dina M.A. Mohamed, Ebtesam A. Ali

**Affiliations:** 1 Lecturer at Basic Science Department, Faculty of Physical Therapy, Cairo University, Cairo, Egypt; 2 Department of Cardiovascular/Respiratory Disorders and Geriatrics, Faculty of Physical Therapy, Cairo University, Cairo, Egypt; 3 Department of Physical Therapy for Women’s Health, Faculty of Physical Therapy, Cairo University, Cairo, Egypt

**Keywords:** Iontophoresis, Magnesium sulphate, Ultrasound, Trapezius muscle, Trigger points

## Abstract

**Background:**

The investigation aimed to assess the impacts of magnesium sulphate (MgSO4) iontophoresis and high-power pain-threshold ultrasound (HPPT-US) on pain, range of motion (ROM), and functional activity in physical therapy students suffering from mechanical cervical pain.

**Methods:**

Typically, 75 males aged 19 to 30 years suffering from mechanical neck pain were enrolled in this investigation. Participants were divided at random into three groups. Group A received iontophoresis plus conventional physical therapy program, Group B received HPPTUS along with conventional therapy, and Group C received conventional therapy only. The outcomes were pain evaluated by visual analog scale (VAS) and Digital Electronic Pressure Algometer, cervical range of motion measured by Myrin gravity reference goniometer, and Arabic Neck disability index (ANDI) evaluate neck function.

**Results:**

The differences within and between groups were detected utilizing a mixed-design multivariate analysis of variance (MANOVA). The within- and between-group analysis of all outcome measures revealed that there were statistically significant differences at post-intervention between high-power ultrasound and conventional group at all variables and also between iontophoresis and conventional group, but there was no statistically significant variation between high-power ultrasound and iontophoresis.

**Conclusion:**

MgSO4 iontophoresis and HPPT-US are effective in decreasing pain, improving neck function, and improving neck ROM in subjects with mechanical neck pain who have active myofascial trigger points (MTrPs) on the upper fibers of the trapezius with no superiority of one over the other.

**Trail registration:**

The study was registered in the Clinical Trials Registry (registration no: NCT05474898) 26/7/2022.

## Introduction

Mechanical neck pain (MNP), alternatively referred to as chronic non-specific neck pain, is a prevalent chronic musculoskeletal condition. MNP is widely acknowledged as a prominent contributor to disability and is regarded as a prevalent condition in the field of clinical practice [1]. Myofascial trigger points (MTrPs) have been theorized to have a role in the pain processes in MNP patients; however, few investigations have incorporated MTrPs treatment for the management of these individuals [2].

Recently, myofascial pain syndrome (MPS) has emerged as a highly prevalent musculoskeletal disorder in clinical settings. It is categorized by the presence of one or more MTrPs [3]. MTrPs are palpable nodules that are hyperirritable and found within the taut bands of skeletal muscle [4]. Practically, it has been observed that approximately 85% of patients who seek medical care at pain clinics exhibit MTrPs [3]. The incidence of the condition is greater in females (54%) compared to males (45%) [5].

Two primary categorizations exist for MTrPs, namely active and latent. Active MTrPs are characterized by persistent pain, referred pain, diminished muscle strength, and decreased muscle elasticity [6]. Latent MTrPs have diagnostic characteristics comparable to active MTrPs but are less severe, and pain is not persistent but appears by assessment [6]. Previous research has examined the impact of various therapeutic techniques on MTrPs, including electrical stimulation, ischemic compression, dry needling, injections, spray and stretch approaches, laser, ultrasound therapy, phonophoresis, and iontophoresis [3].

Iontophoresis is widely regarded as a highly effective therapeutic method for the management of MTrPs. This technique involves the transdermal administration of medications to the body through the application of direct current [7]. Magnesium sulphate (MgSO4) is recognized for its muscle relaxant properties, vasodilation effects, and analgesic capabilities. The investigation conducted by Ibrahim et al. tested the impact of MgSO4 iontophoresis on MTrPs located in the upper fibers of the trapezius muscle (UT). The researchers concluded that this treatment method directly contributes to the improvement of pain levels, neck range of motion (ROM), and neck function in individuals with active MTrPs in the upper fibers of the trapezius muscle [7].

The generation of thermal energy is the primary and recognized outcome of ultrasound (US), a technique widely employed for the management of MPS. The thermogenic impact of US induces a temporary enhancement in the flexibility of dense collagenous constructions, resulting in a reduction in joint stiffness, pain, and muscle spasms, as well as a momentary improvement in circulation [6].

The utilization of high-power pain threshold ultrasound (HPPTUS) involves the direct application of ultrasound waves onto the TrP in a static and intermittent fashion [8]. The potential effects of HPPT-US may also be attributed to the intense stimulation of US, which has been documented to reduce the amplitude of evoked action potentials and produce a concurrent thermal effect [9]. The nociceptor activity within MTrPs may exhibit a decrease in response when subjected to intensive US stimulation. As a result, this reduction in nociceptor activity could potentially cause a decrease in pain intensity and sensitivity. There is a correlation between enhanced neural membrane capacitance and reduced conductivity, which is associated with elevated amplitudes of trans-membrane current. This phenomenon has also been observed in conjunction with higher intensities of US stimulation [10].

Based on current understanding, there appears to be a lack of research examining the impact of MgSO4 iontophoresis compared to HPPTUS on MTrPs. Consequently, the goal of this study was to assess and compare the impacts of MgSO4 iontophoresis and HPPTUS on MTrPs in the upper trapezius muscle among individuals experiencing mechanical neck pain.

## Materials and methods

### Design of the study

The study was a single-blind, randomized, controlled study that ran from August 2022 to November 2022. The research was performed at Cairo University’s Faculty of Physical Therapy’s outpatient clinic. All procedures were performed based on the Helsinki Declaration [11]. The investigation was approved by the Ethical Committee for Human Research at the Faculty of Physical Therapy (reference no.: P. T. REC/012/003552). Before taking part in the study, All the participants provide written informed consent form. The study was registered in the Clinical Trials Registry (registration no: NCT05474898) 26/7/2022.

### Participants

Typically, 75 patients with chronic mechanical neck pain were diagnosed by their physicians. Their age ranged from 19 to 30 years, and they were recruited from the Faculty of Physical Therapy’s undergraduate and postgraduate students. Active MTrPs on the UT muscle bilaterally, pain at rest, local twitch response, jump sign, restricted ROM, and referred pain over the lateral aspect of the UT fibers and superior to the occiput were all inclusion criteria [12, 13].

### Exclusion criteria

latent MTrPs, a history of pain-causing degenerative disorders, fibromyalgia, fractures of the cervical spine, cervical disc hernia, malignancy, had received any therapy in the previous six months and had any contraindication for US therapy [14].

### Randomization

**Fig. 1:**
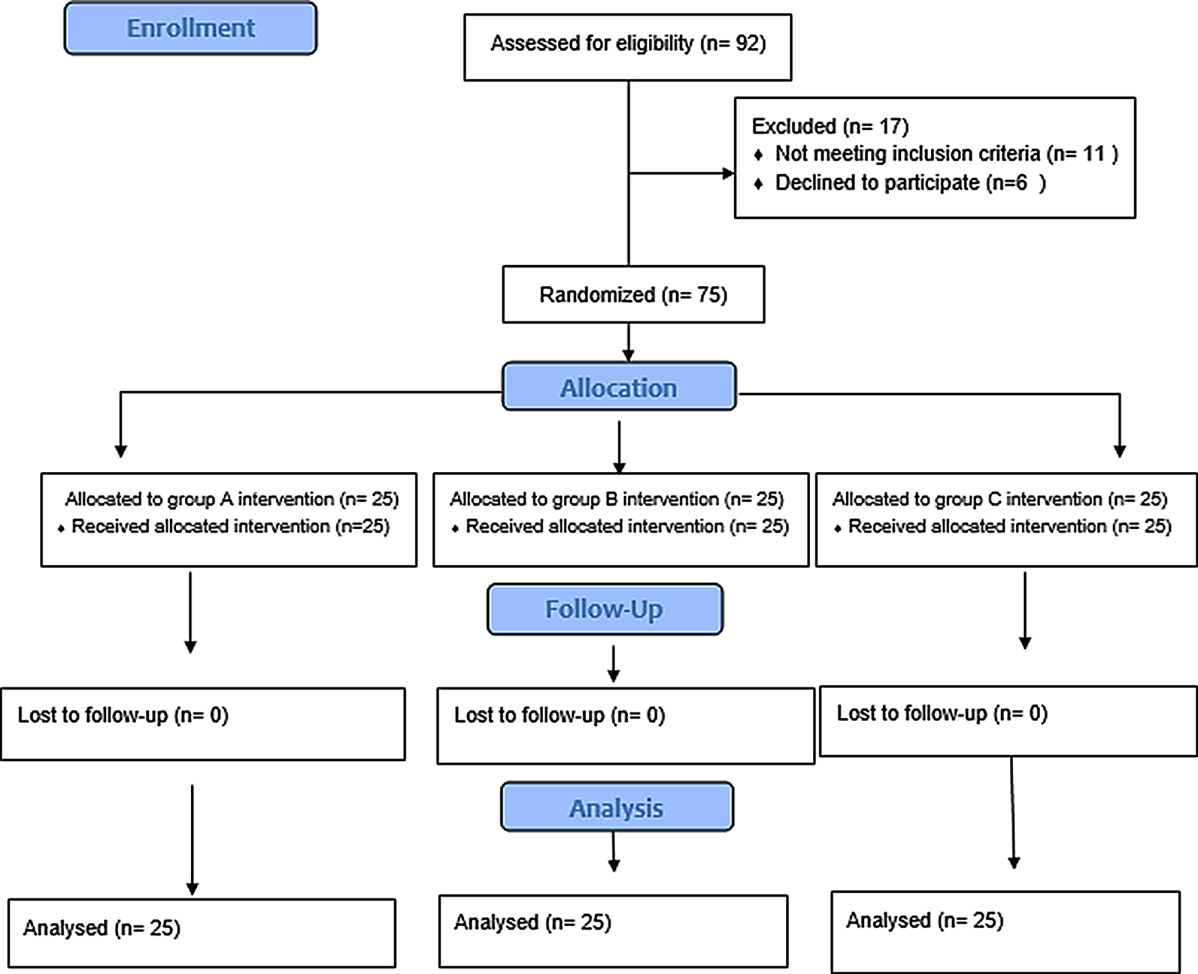
Flowchart for patients in the study (Consolidated Standards of Reporting Trials)

The patients were distributed into three groups: group A underwent iontophoresis plus conventional therapy, Group B received HPPTUS plus conventional treatment, and Group C was the control group (conventional treatment) (Fig. 1**)**. The participants were randomly assigned to one of three groups utilizing a computer-produced random numbers table, and concealed allocation was conducted using sealed opaque envelopes. The second author, who was not aware of the group allocation, measured data collection.

### Outcome measures

All outcome measures were evaluated before the start of intervention and immediately following the completion of treatment.

#### Primary outcome measure

##### Pain intensity assessment

The VAS scale, which is composed of a 10 cm long line on the scale from zero (no pain or discomfort) to 10 (the most intense suffering possible), was utilized. The subject was in a relaxed state [15] and was asked to make a vertical line on the line to detect the level of pain [15].

#### Secondary outcome measures

##### Digital electronic pressure algometer (wagner instruments FDX- Greenwich CT)

The pressure transducer probe of the digital Electronic Pressure Algometer was employed to determine the pressure pain threshold (PPT) of active MTrP tenderness. Numerous researchers have identified pressure algometry validity in the evaluation of MTrPs [16, 17]. The participant was instructed to locate the region of pain at active MTrP while seated. In order to evaluate the PPT in the UT muscle, the PPT value was determined by applying the transducer probe tip perpendicularly over the UT MTrP, holding the pressure in place while gradually increasing it until the subject showed the first indication of discomfort [18].

##### Arabic neck disability index (ANDI)

The Arabic Neck Disability Index, which is a valid and trustworthy tool, was employed to examine neck function [19]. It is distributed into ten categories or classes, and each category has six options (0–5) with a total score of 50. Each subject was requested to select one of six sentences that best defined their function, and the higher score indicated a significant loss of function [20].

##### Myrin gravity reference goniometer (OB goniometer)

The intra-tester reliability of an OB goniometer ranges from fair to high [21]. A Myrin gravity reference goniometer was used to measure the cervical ROM. (Myrin OB Goniometer; OB Rehab Co. Anlic Company, 5-17182 Solana, Sweden). The participant’s neck mobility was assessed while seated in a chair, with his back supported, his feet fully in contact with the ground, and the hip and knee joints perpendicular to it. The strap was then used to secure the device over the head of a participant, who was instructed to move his head to the full, active ROM in each of the six neck movements: The sagittal plane (flexion/extension), frontal plane (right/left lateral flexion), and transverse plane (right/left rotation) [22].

### Therapeutic procedures

#### Iontophoresis

Group A (iontophoresis) subjects underwent MgSO4 iontophoresis utilizing an iontophoretic drug delivery system (Phoresor^®^ II Auto, Model PM850, IOMED) and conventional treatment. Pincer palpation was used to examine and mark UT MTrPs. Using a syringe, MgSO4 at a concentration of 100 mg/cm2 was applied to the active positive electrode. The active electrode was placed directly over the sensitive UT MTrPs. The dispersive electrode was located 6 inches away from the active electrode. The device was set to the required dose of 75 mA/min, and the current intensity was slowly raised, varying from 2 to 4 mA, according to the subject’s sensitivity. The device independently computed the necessary duration for the designated dosage, and the treatment protocol was administered biweekly over four weeks [23].

#### High power pain threshold ultrasound (HPPT-US)

Group (B) received HPPT-US in addition to conventional treatment. Subjects received HPPT-US using a Med Serve system (England NN114HE, Prosound/ULS-1000, S/N: U0547). The probe was fixed over the UT trigger point, and the US dose was elevated until the participant felt intolerable pain. The probe was held fixed for 3 s at the dose that caused intolerable pain, and then the dose was decreased by half. At this dosing regimen, the probe was moved in a circular pattern over the UT trigger point and the nearby areas for 15 s before the dose was increased a second time in the same manner. For 3 s, the maximum dose that could be given to the patients ranged from 1.5 to 2.5 W/cm2 [24, 25]. The same procedure was used three times more before the treatment session finished. The intervention was conducted two times per week for four weeks.

### Conventional treatment

Group (C) received stretching and strengthening protocol, as well as stretching exercises for the upper fibers of the trapezius muscle, was applied for 15–20 s and was conducted three times. Consequently, an isometric strengthening exercise was applied with maximum resistance for head extension and neck side bending. Each position was maintained for 10–15 s and performed five times [26]. The treatment was performed twice per week for four weeks.

### Power analysis

Using the results of the pilot study, the sample size was established prior to the trial’s start, with ten people in each group. F tests, MANOVA, repeated measures, and between-interaction analyses on the main outcome variable (pain intensity) were executed using G*POWER (version 3.1.9.2; Franz Faul, Universitat Kiel, Germany). The effect size was set at 0.29, the alpha level at 0.05, and the beta level at 0.2. These criteria indicated that the sample size should have been 57; however, due to dropouts, the actual number was increased by 30–75%.

### Statistical analysis

Each variable was checked for normality using the Shapiro-Wilk test, and they were all found to be normalized. The physical features of the patients were compared between the three groups using a one-way analysis of variance (ANOVA). A mixed multivariate analysis of variance (MANOVA) was used to investigate the effects of treatments, time, and the interaction between time and treatment. In cases where the MANOVA revealed statistically significant effects, a follow-up univariate ANOVA is used. Several pairwise comparisons using the Bonferronie correction were conducted to prevent type 1 errors. A partial eta square (*η*) was used to measure how much each group differed. The SPSS version 23 was used for all analyses (IBM Corp., New York, USA).

## Results

### Physical characteristics

ANOVA revealed that age, weight, height, and body mass index (BMI) were not statistically significantly different from one another (Table 1).

**Table 1:** Patient’s physical characteristics

	Mean ± SD			*p*-value
	High power ultrasound	iontophoresis	Control	
Age: (years)	21.72 ± 3.67	20.92 ± 2.1	22.36 ± 2.95	0.24 ^**^
Weight: (kg)	64.28 ± 7.54	64.76 ± 6.57	65.28 ± 5.42	0.86 ^**^
Height: (cm)	163.28 ± 6.11	164.52 ± 5.38	166.32 ± 9.07	0.31 ^**^
BMI: (kg/m^*2*^)	24.1 ± 2.18	23.89 ± 1.82	23.7 ± 2.41	0.83 ^**^

^**^: no significance difference; SD: standard deviation; p-value: significance level; BMI: body mass index

MANOVA indicated a statistically significant variation between treatments as Wilks’ Lambda (ʎ) = 0.23, f = 6.83, *p* = 0.0001, and n^2^=0.52. Also, there were significant differences at the time as ʎ = 0.04, f = 167.8, *p* = 0.0001, and n^2^ = 0.96. Finally, there were significant interactions between treatment and time as ʎ = 0.19, f = 7.87, *p* = 0.0001, and n^2^ =0.55.

After intervention, there were statistically significant effects regarding follow-up univariate ANOVA for pain intensity by VAS as *p* < 0.0001, f _(2,72)_ = 46.95 and n^2^ =0.57; for right PPT, *p* < 0.0001, f _(2,72)_ = 23.97 and n^2^ =0.4; for left PPT, *p* < 0.0001, f _(2,72)_ = 41.44 and n^2^ =0.53; for neck disability by ANDI, *p* < 0.0001, f _(2,72)_ = 23.15 and n^2^ =0.39; for flexion, *p* < 0.0001, f _(2,72)_ = 29.89 and n^2^ =0.45; for extension, *p* < 0.0001, f _(2,72)_ = 26.93 and n^2^ =0.43; for side bending right side, *p* < 0.0001, f _(2,72)_ = 48.25 and n^2^ =0.57, for side bending left side, *p* < 0.0001, f _(2,72)_ = 57.74 and n^2^ =0.62, for rotation right, *p* < 0.0001, f _(2,72)_ = 74.83 and n^2^ =0.67, for rotation left, *p* < 0.0001, f _(2,72)_ = 78.7 and n^2^ =0.69 (Table 2).

**Table 2:** Within and between group analysis

Variables	High power ultrasound	iontophoresis	control	*p*-value between	f-value Between	n^2^
VAS						
Baseline	7.68 ± 0.75	7.2 ± 1.15	7.44 ± 0.96	0.23 ^**^	1.53	0.04
Post-intervention	1.68 ± 0.3	1.88 ± 0.66	4.52 ± 1.66	0.0001 ^*^	46.95	0.57
p-value (within)	0.0001 ^*^	0.0001 ^*^	0.0001 ^*^			
MD	6	5.32	2.92			
95% CI	5.4 to 6.6	4.7 to 5.9	2.3 to 3.5			
Right PPT						
Baseline	0.62 ± 0.25	0.61 ± 0.19	0.59 ± 0.26	0.89 ^**^	0.11	0.03
Post-intervention	1.66 ± 0.35	1.81 ± 0.77	0.9 ± 0.15	0.0001 ^*^	23.97	0.4
p-value	0.0001 ^*^	0.0001 ^*^	0.0004 ^*^			
MD	-1.035	-1.19	-0.31			
95% CI	-1.24 to -0.87	-1.4 to -0.99	-0.52 to -0.1			
Left PPT						
Baseline	0.58 ± 0.25	0.63 ± 0.24	0.56 ± 0.21	0.63 ^**^	0.47	0.01
Post-intervention	1.83 ± 0.34	1.75 ± 0.35	1.01 ± 0.36	0.0001 ^*^	41.44	0.53
p-value (within-group)	0.0001 ^*^	0.0001 ^*^	0.0001 ^*^			
MD	-1.24	-1.12	-0.45			
95% CI	-1.41 to -1.08	-1.29 to -0.96	-0.61 to -0.28			
Neck disability (ANDI)						
Baseline	26.8 ± 2.77	25.08 ± 5.47	26 ± 3.33	0.32 ^**^	1.14	0.03
Post-intervention	10.4 ± 1.44	9.24 ± 3.52	16.28 ± 5.62	0.0001 ^*^	23.15	0.39
p-value	0.0001 ^*^	0.0001 ^*^	0.0001 ^*^			
MD	16.4	15.84	9.72			
95% CI	14.38 to 18.41	13.82 to 17.85	7.7 to 11.73			
Flexion						
Baseline	47.4 ± 10.13	47.5 ± 10.12	45.32 ± 5.94	0.64 ^**^	0.45	0.01
Post-intervention	60.04 ± 6.3	61.08 ± 6.29	50 ± 3.82	0.02 ^*^	29.89	0.45
p-value	0.0001 ^*^	0.0007 ^*^	0.005 ^*^			
MD	-12.64	-13.68	-4.68			
95% CI	-15.84 to -9.43	-16.88 to -10.47	-7.88 to -1.47			
Extension						
Baseline	46.96 ± 8.22	47.16 ± 7.97	45.96 ± 6.9	0.084 ^**^	0.17	0.05
Post-intervention	63.08 ± 8.76	63.88 ± 8.17	49.24 ± 6.72	0.0001 ^*^	26.94	0.43
p-value	0.0001 ^*^	0.0001 ^*^	0.03 ^*^			
MD	-16.12	-16.72	-3.28			
95% CI	-19.1 to -13.14	-19.7 to -13.73	-6.26 to -0.29			
Right side bending						
Baseline	34.04 ± 4.43	34.52 ± 4.17	33.4 ± 4.51	0.66^**^	0.41	0.01
Post-intervention	43.84 ± 2.07	43.6 ± 2.1	36.76 ± 4.16	0.0001 ^*^	48.25	0.57
p-value	0.0001 ^*^	0.0002 ^*^	0.001 ^*^			
MD	-9.8	-9.08	-3.36			
95% CI	-11.8 to -7.8	-11.32 to -7.32	-5.36 to -1.36			
left side bending						
Baseline	33.12 ± 5.06	33.32 ± 3.97	32.24 ± 3.03	0.62^**^	0.49	0.01
Post-intervention	44.12 ± 1.74	44.04 ± 1.74	36.68 ± 4.2	0.0001 ^*^	57.74	0.62
p-value	0.0001 ^*^	0.0002 ^*^	0.0001 ^*^			
MD	-11	-10.72	-4.44			
95% CI	-12.8 to -9.2	-12.52 to -8.92	-6.24 to -2.64			
Right rotation						
Baseline	46.68 ± 8.04	47.28 ± 8.33	46.68 ± 4.8	0.94^**^	0.06	0.002
Post-intervention	70.72 ± 5.4	69.08 ± 5.65	53.4 ± 5.51	0.0001 ^*^	74.83	0.67
p-value	0.0001 ^*^	0.0001 ^*^	0.0001 ^*^			
MD	-24.04	-21.8	-6.72			
95% CI	-27.54 to -20.54	-25.3 to -18.3	-10.22 to -3.22			
Left rotation						
Baseline	49.04 ± 8.61	47.72 ± 7.02	47 ± 5.2	0.59^**^	0.53	0.01
Post-intervention	71.24 ± 3.89	72.32 ± 4.2	55.48 ± 7.2	0.0001 ^*^	74.7	0.69
p-value	0.0001 ^*^	0.0001 ^*^	0.0001 ^*^			
MD	-22.2	-24.6	-8.48			
95% CI	-25.53 to -18.86	-27.94 to -21.26	-11.82 to -5.14			

^**^: no significance difference; ^*^: significant difference; SD: standard deviation; p-value: significance level set at 0.05; PPT: pressure pain threshold; ANDI: Arabic neck disability index; VAS: Visual Analogue Scale; CI: confidence interval; MD: mean difference; n^2^: Partial Eta Square

### Within and between group’s analysis

Pairwise comparisons indicated statistically significant variations between the starting point and post-intervention for all variables in high-power ultrasound, iontophoresis, and control group as p-value < 0.05, **as in** Table 2. Between groups analysis at baseline, there were no statistically significant variations (Table 2); however, there were statistically significant changes at post-intervention between high power ultrasound and control group at all variables and also between iontophoresis and control group, but there were no statistically significant alterations between high power ultrasound and iontophoresis at all variables except (Table 3).

**Table 3:** Multiple pairwise comparison

variables	High-power ultrasound vs. iontophoresis (MD)(CI (95%)/ *p*-value)		High-power ultrasound vs. control (MD)(CI (95%)/ *p*-value)		iontophoresis vs. control (MD)(CI (95%)/ *p*-value)	
VAS	-0.2(-1 to 0.6)	0.99 ^**^	-2.8(-3.64 to -2.04)	0.0001 ^*^	-2.64 (-3.44 to -1.84)	0.0001 ^*^
Right PPT	-0.15 (-0.5 to 0.2)	0.84 ^**^	0.76(0.41 to 1.1)	0.0001 ^*^	0.91 (0.56 to 1.25)	0.0001 ^*^
Left PPT	0.08(-0.17 to 0.32)	0.99^**^	0.82(0.57 to 1.06)	0.0001^*^	0.74 (0.5 to 0.99)	0.0001^*^
Neck disability (ANDI)	1.16(-1.56 to 3.88)	0.89^**^	-5.88(-8.6 to -3.16)	0.0001 ^*^	-7(-9.75 to -4.32)	0.0001 ^*^
flexion	-0.04(-4.92 to 2.84)	0.99^**^	10(6.16 to 13.92)	0.0001 ^*^	11.08(7.2 to 14.96)	0.0001 ^*^
Extension	-0.8(-6.3 to 4.7)	0.99^**^	13.84(8.34 to 19.3)	0.0001^*^	14.64 (9.14 to 20.14)	0.0001 ^*^
Right side bending	0.24(-2.04 to 2.12)	0.99^**^	7.08(5.04 to 9.12)	0.0001 ^*^	6.84 (5.04 to 9.12)	0.0001 ^*^
left side bending	0.08(-1.87 to 2.03)	0.99^**^	7.44(5.5 to 9.39)	0.0001^*^	7.36 (5.41 to 9.3)	0.0001 ^*^
Right rotation	1.64(-2.19 to 5.47)	0.89^**^	17.32(13.5 to 21.15)	0.0001 ^*^	15.68 (11.84 to 19.51)	0.0001 ^*^
Left rotation	-1.08(-4.76 to 2.6)	0.99^**^	15.76(12.07 to 19.44)	0.0001 ^*^	16.84 (13.16 to 20.52)	0.0001 ^*^

^**^: no significance difference; ^*^: significant difference; SD: standard deviation; p-value: significance level set at 0.05; PPT: pressure pain threshold; ANDI: Arabic neck disability index; VAS: Visual Analogue Scale; CI: confidence interval; MD: mean difference

## Discussion

This investigation aimed to compare the impact of MgSO4 iontophoresis with that of HPPT-US in participants with mechanical neck pain with UT MTrPs. The results showed reduced pain, improved neck function, and improved neck ROM in both MgSO4 iontophoresis and HPPT-US groups, with no statistically significant difference. Also, the minimal clinical importance difference for VAS was 3.1 [27]. So, there was no clinically important difference between MgSO4 iontophoresis and HPPT-US in VAS measurement.

The prevailing theory regarding the etiology of MTrPs is the energy crisis theory. According to this theory, MTrPs develop as a consequence of repetitive microtrauma or macrotrauma to muscle fibers. This trauma leads to an unnecessary release of calcium and a sustained contraction of the sarcomeres, which subsequently impairs blood and oxygen supply to the affected area. Consequently, there is an inadequate synthesis of adenosine triphosphate (ATP), a crucial molecule for initiating muscle relaxation. The persistent contraction of muscles results in the accumulation of metabolic waste products, thereby resulting in the sensation of pain [28].

Moreover, according to the motor endplate theory, the development of MTrPs can be attributed to the atypical and excessive production of acetylcholine from the motor end plate, even during periods of muscle relaxation. This leads to a deliberate shortening of sarcomeres and the subsequent development of contraction knots within the muscle fibers [29]. The aforementioned theories have the potential to provide valuable insights into the mechanisms underlying the therapeutic effects of both MgSO4 iontophoresis and HPPT-US techniques in the management of MTrPs.

One probable reason for the observed enhancement in the group receiving MgSO4 iontophoresis treatment is that the reduction in pain levels may be attributed to the vasodilatory properties of MgSO4. This vasodilation effect can counteract vasoconstriction in various vascular beds, thereby improving blood supply to the trigger point and eliminating the irritating substance responsible for pain [30, 31]. Additionally, the improvement in neck function may be attributed to the connection between neck pain and disability, as investigated by Fejer and Hartvigsen [32]. Their research suggests a moderate association between pain and disability, with neck disability increasing linearly as the number of pain locations and pain-inducing factors increases. The findings of the present investigation show that the reduction of pain has a positive impact on neck function. This enhancement in neck ROM can be related to the muscle relaxation properties of MgSO4, which acts by reducing the release of acetylcholine at the myoneural junction. This mechanism effectively blocks peripheral neuromuscular transmissions, resulting in the suppression of skeletal muscle contractions [33].

The findings of the MgSO4 iontophoresis group align with the conclusions drawn by Ibrahim et al. (2021), indicating that MgSO4 iontophoresis is efficacious in enhancing pain levels, neck range of motion, and neck function in relation to MTrPs of UT muscle [7]. Furthermore, the results presented in this research align with the findings reported by Teslim et al. [23], which indicated a significant reduction in spasticity of the biceps brachii muscle in stroke patients after using MgSO4 iontophoresis.

Contrasted to our results, an investigation conducted by **Evans et al. (2001)** indicated that using lidocaine iontophoresis with a 1% concentration of lidocaine is not beneficial in treating MTrPs. The potential source of this discrepancy between the two investigations may stem from variations in the administered drug [34].

One potential reason for the observed enhancement in the HPPT-US group might be attributed to the elevated intensity of US stimulation, which has been shown to decrease the amplitude of evoked action potentials and have an extra thermal impact [9]. The nociceptor activity inside MTrPs may exhibit a reduction when subjected to extensive US stimulation, resulting in a potential decrease in pain intensity and sensitivity. The relationship between enhanced neural membrane capacitance and reduced conductivity is associated with elevated amplitudes of trans-membrane current, a phenomenon that has also been seen in conjunction with greater intensities in the US [10].

The findings in the HPPT-US group align with the results reported by Elhafez et al. (2020), who examined the effect of HPPT-US on pain and myoelectric activities of the UT muscle. Their study demonstrated that HPPT-US effectively reduces pain levels and restores resting myoelectric activities of the UT muscle [35]. Furthermore, our findings align with the results reported by **Koca et al. (2014)**, which revealed the efficacy of HPPT-US as a therapy approach for MTrPs based on assessments of VAS score and neck active lateral bending ROM [36].

Furthermore, our findings correspond with the outcomes reported by **Unalan et al. (2011)** in a study that aimed to compare the efficacy of HPPT-US therapy and local anesthetic injection in individuals with active myofascial trigger points in the upper trapezius muscle. The investigation demonstrated that both local anesthetic injection and HPPT-US therapy led to improvements in pain and ROM scores in individuals with MPS [8].

In contrast to the results obtained in our study involving the HPPT-US group, Gam et al. (1998) performed a randomized controlled trial to investigate the impact of ultrasound treatment joined with massage and exercise on myofascial trigger points. Their findings revealed that ultrasound did not lead to a reduction in pain, whereas massage and exercise appeared to decrease the quantity and intensity of myofascial trigger points. The observed disparity in findings between the current study and prior research might perhaps be due to the use of distinct parameters within the US [37].

The observed enhancement in the control group may be caused by the quick hypoalgesic impact of isometric workouts when performed with stretching exercises [38]. Stretching exercises can also aid in the relaxation of muscle spasms. This approach operates by targeting the viscoelastic characteristics of the muscles to induce relaxation. Conducting a continuous external load gradually to a shortened muscle causes stretching and improves the flexibility of the target muscle [39].

Finally, we failed to find significant difference between both modalities may be due to some limitation in this study as the treatment period is short and no follow up was applied, they differ in their mechanism of action, benefits and limitation.

The intensity of HPPT-US therapy varies from patient to patient based on their individual pain levels, making standardization difficult. Attempting to standardize the treatment may result in some patients receiving inadequate dose while others may be over-treated. In contrast, the iontophoresis group allows for a standardized dose to be set on the device, with intensity adjusted based on the patient’s sensitivity.

The HPPT-US technique requires the therapist to have additional experience and strong communication and concentration from both the patient and therapist [8]. However, iontophoresis is a simple technique to administer, some patients may experience skin allergic reactions with prolonged exposure to direct electric current.

Moreover, the application time for HPPT-US was relatively short, with a total duration of about one minute, demonstrating both the efficiency and cost-effectiveness of the treatment. In contrast, iontophoresis may require 20 min or more to administer the necessary dose.

HPPTUS should not be used on TrPs that are located near bones or nerves to prevent potential damage to these structures. It is also important to differentiate between pain caused by a TrP and discomfort from excessive heating, particularly if ultrasound is applied for more than three seconds [8]. On the other hand, iontophoresis can be safely administered near bones, nerves, and soft tissues. Clinicians must consider the patient’s specific condition, medical history, and the desired therapeutic effect when choosing between these techniques. Both have their unique strengths, and tailoring the treatment to the patient’s needs will yield the best outcomes.

### Study limitations

The study’s limitation was focusing on short-term treatment effects. Longer-term follow-up is required in addition to comparisons with other interventions.

## Conclusion

MgSO4 iontophoresis and HPPT-US are effective in decreasing pain, improving neck function, and improving neck ROM in subjects with mechanical neck pain who have active MTrPs on the upper fibers of the trapezius with no superiority of one over the other.

## Data Availability

No datasets were generated or analysed during the current study.

## Abbreviations

MgSO4: Magnesium Sulphate
HPPT-US: High-Power Pain-Threshold Ultrasound
ROM: Range Of Motion
VAS: Visual Analog Scale
ANDI: Arabic Neck disability index
MTrPs: Myofascial Trigger Points
MNP: Mechanical neck pain
MPS: Myofascial Pain Syndrome
PPT: Pressure Pain Threshold
